# Modulation of Invasive Phenotype by Interstitial Pressure-Driven Convection in Aggregates of Human Breast Cancer Cells

**DOI:** 10.1371/journal.pone.0045191

**Published:** 2012-09-18

**Authors:** Joe Tien, James G. Truslow, Celeste M. Nelson

**Affiliations:** 1 Department of Biomedical Engineering, Boston University, Boston, Massachusetts, United States of America; 2 Department of Chemical and Biological Engineering and Department of Molecular Biology, Princeton University, Princeton, New Jersey, United States of America; Cedars-Sinai Medical Center, United States of America

## Abstract

This paper reports the effect of elevated pressure on the invasive phenotype of patterned three-dimensional (3D) aggregates of MDA-MB-231 human breast cancer cells. We found that the directionality of the interstitial pressure profile altered the frequency of invasion by cells located at the surface of an aggregate. In particular, application of pressure at one end of an aggregate suppressed invasion at the opposite end. Experimental alteration of the configuration of cell aggregates and computational modeling of the resulting flow and solute concentration profiles revealed that elevated pressure inhibited invasion by altering the chemical composition of the interstitial fluid near the surface of the aggregate. Our data reveal a link between hydrostatic pressure, interstitial convection, and invasion.

## Introduction

How physical forces affect the invasive phenotype of human cancer cells remains unclear [1]–[3]. Among the possible physical signals, mechanical rigidity, interstitial flow, and direct mechanical stress have been shown to influence cell invasion to varying degrees in vivo and in culture [4]–[6]. For instance, Swartz and colleagues have found that interstitial flow, which is driven by a gradient in interstitial fluid pressure, can lead to migration of single cells via “autologous chemotaxis” [5]. This downstream migration occurs via polarization of an autocrine chemotactic factor (e.g., CCL21 released by CCR7-expressing cells), so that a cell experiences a positive concentration gradient in the direction of flow. In contrast, Kamm and colleagues demonstrated that interstitial flow can lead to upstream migration of single cells in regions of high cell density [7]. They speculated that the upstream end of a cell may experience greater adhesion stresses under flow, and this polarized distribution of stress may lead to local mechanotransduction that results in migration towards regions of higher pressure. Moreover, a recent study has suggested that compressive stresses can induce invasion from two-dimensional clusters of cancer cells [6]. These studies suggest that the migratory response of cancer cells to physical signals may depend on the context in which the signals are presented (e.g., low versus high cell density, two- versus three-dimensional culture) [8].

The objective of the current paper is to elucidate how pressure affects invasion from three-dimensional aggregates of human breast cancer cells. We wished to better understand the physical factors that lead to initial invasion from a cell aggregate, as might be seen in the transition from a pre-invasive to invasive tumor. We elected to study these issues in a cell culture model that allowed independent control of interstitial pressure at specific locations along a millimeter-scale packed aggregate of MDA-MB-231 human breast cancer cells. By varying the configuration of the culture model, we isolated the direct and indirect effects of mechanical signals on invasion. In this model system, elevated pressure suppressed invasion at the opposite end where pressure was lower, and pressure-induced convection of soluble factors was responsible for changes in invasive phenotype.

## Materials and Methods

### Cell Culture

We cultured MDA-MB-231 human breast cancer cells (ATCC) in DMEM/F12 media (Hyclone) with 10% FBS (Atlanta Biologicals) and 50 µg/mL gentamicin, and passaged them at a 1∶4 ratio every 3–4 days up to passage nine.

### Formation of Cell Aggregates

**Figure 1:**
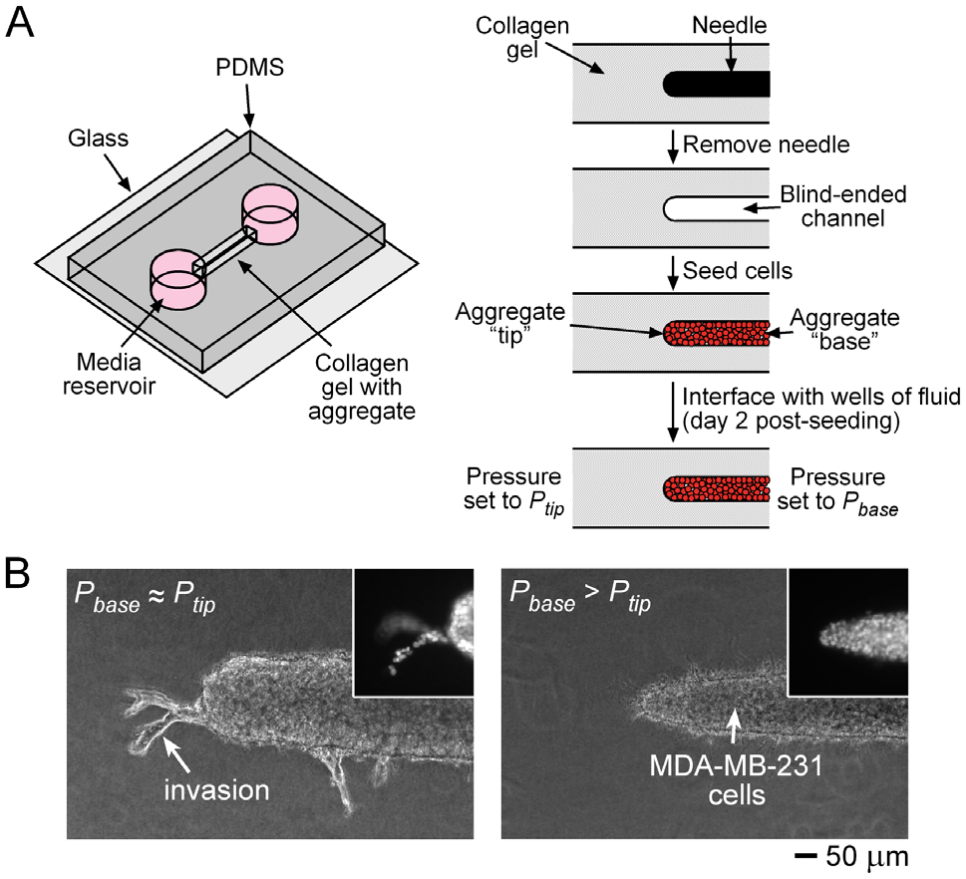
Formation of 3D micropatterned aggregates of MDA-MB-231 cells. (A) Schematic diagram of the completed experimental setup (*left*) and the procedure used to form single aggregates (*right*). Each aggregate consisted of a “base” that ended in a “tip” in a type I collagen gel. (B) Representative phase-contrast and fluorescence (*insets*) images of Hoechst-stained tips for *P_base_* equal to or greater than *P_tip_*, seven days after establishing the pressure set-points.

To form single tumor cell aggregates (*n* = 678), we adapted a needle-based approach to mold blind-ended cavities in type I collagen gels within a chamber of polydimethylsiloxane (PDMS) (Fig. 1A) [9]. We gelled a solution of liquid collagen (4 mg/mL, pH 9 from bovine dermis; Koken) around 120-µm-diameter needles (Seirin) for ∼20 min at 37°C, removed the needles, and seeded MDA-MB-231 cells as a concentrated suspension (one 35-mm-dish in 1 mL media) into the resulting cavities. The dimensions of the gel were ∼1×1×9 mm^3^, and the aggregates were 4–4.5 mm long. We then added 20–50 µL media to both ends of the gel to ensure that the gel remained well-hydrated. One end (the “tip”) of an aggregate was located near the midpoint of the gel, while the other end (the “base”) was at the mouth of the cavity and thus in fluidic contact with media. Two days after seeding cells, we established interstitial pressure profiles by setting the heights of media located in reservoirs at the aggregate base (*P_base_*) and at the opposite end of the collagen gel (*P_tip_*). For samples with *P_base_*>*P_tip_* or *P_base_*<*P_tip_*, we set the lower pressure to be 0 cm H_2_O, and the higher pressure to be 0.4–1.6 cm H_2_O; we generated the positive pressure by stacking ∼4-mm-thick PDMS ring-shaped spacers over an open side and filling the resulting well with media. For samples with *P_base_* ♦ *P_tip_* or *P_base_* 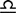 *P_tip_*, we set both pressures to be 0.15 cm H_2_O and then added a slight excess of media to one side. We replenished media every twelve hours to maintain the pressure set-points. In some samples, we combined F12 media with low-glucose DMEM (Invitrogen) to obtain media with a glucose content of 2.4 mg/mL (with or without 0.7 mg/mL lactic acid) or 3.1 mg/mL.

We used similar methods to form opposing aggregates (*n* = 33), single aggregates that faced an empty cavity (*n* = 39), or single aggregates in a T-shaped gel (*n* = 45). For the first two cases, the lengths of the aggregates or empty cavities were ∼3.5 mm, with a ∼3.5 mm spacing between their tips. Aggregates in a T-shaped gel were ∼6 mm long; their tips were ∼3 mm and ∼4.5 mm from the upstream and downstream wells, respectively. We typically set the downstream pressure (*P_right_* or *P_lower_*) to be 0 cm H_2_O and the upstream pressure (*P_left_* or *P_upper_*) to be 0.4–1.6 cm H_2_O. In some of the opposing aggregates, we set *P_left_* and *P_right_* to be 0.15 cm H_2_O and added a slight excess of media to one side.

We collected conditioned media from the downstream reservoir of single aggregates grown under *P_base_*>*P_tip_* (20–100 µL per sample per day, depending on the magnitude of the pressure difference), and used the fractions as is, supplemented with 10% FBS, or dialyzed overnight against fresh culture media using a 3.5 kDa cutoff filter (D-Tube Dialyzer Midi Kit; EMD Chemicals).

### Invasion Assay

Seven days after establishing interstitial pressure profiles (nine days after seeding cells), we treated samples with media containing Hoechst 33342 (1∶1000; Invitrogen) and visualized them under UV illumination or phase-contrast optics using a Hamamatsu Orca CCD camera attached to a Nikon Ti-U microscope using a Plan Fluor 10×/0.30 NA objective. We measured lengths of invasions using HC Image version 2.2 (Hamamatsu). In some samples with *P_base_* 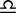 *P_tip_*, we changed the pressure set-points nine days after seeding to *P_base_*>*P_tip_* and performed the invasion assay after an additional five days. In some samples with *P_base_*>*P_tip_*, we changed the pressure set-points seven days after seeding to *P_base_* ♦ *P_tip_* or *P_base_* 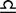 *P_tip_* and performed the invasion assay after an additional seven days.

### Cell Viability Assay

To analyze cell viability within aggregates, we stained single aggregates 4–6 days after establishing interstitial pressure profiles (6–8 days after seeding cells) with 4 µM ethidium homodimer-1 (Invitrogen) and Hoechst 33342 at 1∶1000 dilution for ∼2 hours before imaging. Nuclei that stained positively with ethidium homodimer were considered non-viable.

### Measurement of Hydraulic Permeabilities and Glucose Concentrations

We calculated the hydraulic permeability of the collagen gels by forming ∼1×1×8 mm^3^ blocks of gel in a PDMS channel, applying a ∼1 cm H_2_O pressure difference across the ends, measuring the resulting flow rate of media, and applying Darcy’s Law for flow through porous solids. We used a similar procedure to measure the permeability of MDA-MB-231 aggregates, except the geometry consisted of up to a dozen ∼180-µm-diameter, 6-mm-long cylinders in parallel. We used a glucose oxidase-based colorimetric assay kit (Sigma) to measure glucose concentrations in conditioned media.

### Computational Modeling

We used COMSOL Multiphysics version 3.5a to analyze fluid and solute transport in 3D models that reproduced the various experimental geometries. Darcy’s Law (using the measured gel and cell aggregate permeabilities) and a reaction-convection-diffusion equation described fluid and solute transport, respectively; we omitted the reaction term in the gel. Since the nature of relevant invasion-altering solutes is largely unknown, we performed a parametric sweep of the solute diffusion coefficient (1–100 µm^2^/s) and reaction rates. To model a solute-producing aggregate, we assumed zeroth-order production rates of 0.3–3×10^−10^ mol/cm^3^/s. To model a solute-consuming aggregate, we assumed first-order consumption rate constants of 1/3000–1/300 s^−1^, with an input starting concentration of 10^−7^ mol/cm^3^. We chose these values to mimic representative data for small molecular weight solutes in solid tumors and in DMEM/F12 media: ∼100 µm^2^/s for the diffusion coefficient for a ∼1-nm-radius solute [10], ∼1/2500 s^−1^ for the glycolytic rate constant [11], and 10^−7^–10^−6^ mol/cm^3^ for the concentrations of amino acids. For each model, we checked mesh convergence by doubling the degrees of freedom (up to five million) until the concentration at the aggregate tip changed by ≤5×10^−10^ mol/cm^3^. Solute concentration gradients were computed normal to the aggregate surface; a positive value indicates a higher concentration in the gel than at the cell surface.

To obtain the contribution of interstitial flow to the adhesion stress normal to the cell-gel interface, we used the calculated flow to obtain the pressure on a model cell (a sphere of radius 10 µm) [12]. A negative value indicates a compressive stress (i.e., the cell is “pushed” into the gel).

### Statistical Analysis

Comparisons of invasion frequencies used Fisher’s exact test or (for paired values from the upper and lower halves of aggregates in T-shaped gels) Wilcoxon’s signed rank test. Comparisons of non-categorical data used Mann-Whitney U test. We used Prism version 5 (GraphPad) for all statistical tests, and considered a *p* value less than 0.05 divided by the number of comparisons to indicate a statistically significant difference. Data are presented as means ± SD.

## Results

### Elevated Pressure at the Aggregate Base Suppresses Invasion from the Aggregate Tip

We developed a technique to apply defined pressures across three-dimensional (3D) aggregates of breast cancer cells (Fig. 1A). We used 120-µm-diameter needles as templates to form blind-ended cavities within type I collagen gels. Introduction of a dense suspension of MDA-MB-231 cells at the open end caused convective transport of cells into the cavity. This process resulted in the formation of a packed cell aggregate that we could monitor repeatedly for invasion near the end (the “tip”) that faced the gel; we note that collagen gel completely surrounded the tips of the aggregates, so that we did not need to consider invasion along an interface (e.g., between the collagen gel and an underlying surface). By linking the ends of the collagen gel to fluid reservoirs, we controlled the pressure profile within the aggregate. In some of the samples, the pressure difference *P_base_* − *P_tip_* ranged from 0.4 to 1.6 cm H_2_O, and interstitial flow velocities were on the order of 1 µm/s (based on the average flow rates of 20–100 µL/day). In other samples, we set *P_base_* to be nearly equal to *P_tip_*, with an average pressure difference of 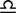
0.1 cm H_2_O and correspondingly smaller interstitial flows.

**Figure 2:**
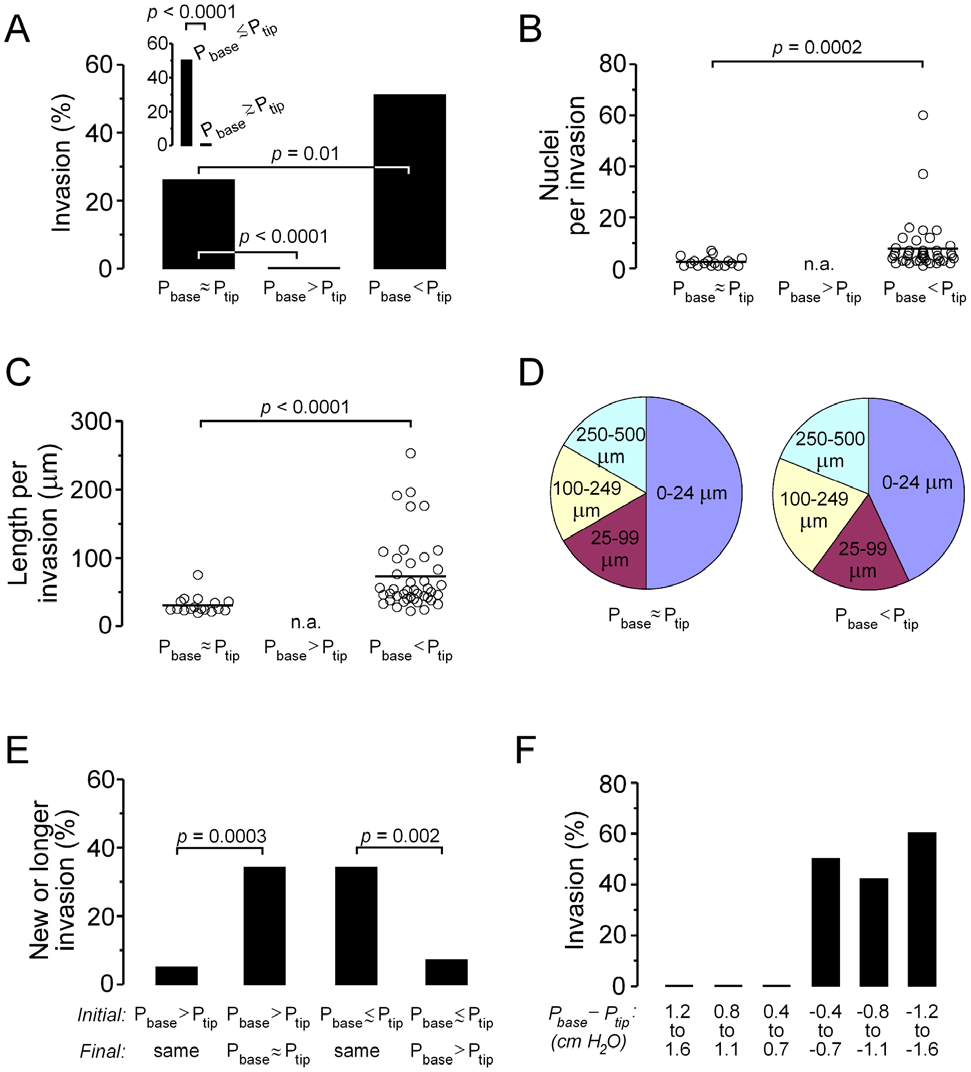
Suppression of invasion by elevated *P_base_* relative to *P_tip_*. (A–C) Invasion frequency (A), number of nuclei per invasive protrusion (“invasion”) (B), and length per invasion (C) for the three pressure conditions. In (A), *n* = 57, 56, and 51 for *P_base_* ≈ *P_tip_*, *P_base_*>*P_tip_*, and *P_base_*<*P_tip_*, respectively. (D) Distribution of invasive protrusions as a function of distance from the aggregate tip. (E) Frequency of new invasion or extension of pre-existing invasive protrusion for aggregates that were switched from *P_base_*>*P_tip_* to *P_base_* ≈ *P_tip_* (*n* = 65) or left unchanged (*n* = 42), or from *P_base_* 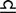 *P_tip_* to *P_base_*>*P_tip_* (*n* = 45) or left unchanged (*n* = 38). (F) Invasion frequency as a function of *P_base_* − *P_tip_* (*n* = 13, 27, 16, 14, 22, and 15 for *P_base_* − *P_tip_* of 1.2 to 1.6 cm H_2_O, 0.8 to 1.1 cm H_2_O, 0.4 to 0.7 cm H_2_O, −0.4 to −0.7 cm H_2_O, −0.8 to −1.1 cm H_2_O, and −1.2 to −1.6 cm H_2_O, respectively).

As expected for an invasive cell line such as MDA-MB-231, aggregates that were subjected to a control pressure profile (i.e., *P_base_* ≈ *P_tip_*, with little to no interstitial pressure gradient) invaded robustly (Fig. 1B); roughly one-quarter of samples invaded after nine days in culture (Fig. 2A). These invasions were typically multicellular (Fig. 1B, inset and 2B), and often extended many tens of micrometers from the surface of the aggregate (Fig. 2C). The collective nature of the invasions is consistent with the findings of Friedl and colleagues for MDA-MB-231 cells [13], [14]. The invasions concentrated near the aggregate tip, with nearly half originating from within 25 µm of the tip (Fig. 2D).

Aggregates that were subjected to elevated pressure at their base (i.e., *P_base_*>*P_tip_*) did not invade [Fig. 1B; *p*<0.0001 compared with control (Fig. 2A)]. Instead, the tumor cells sent forth numerous fine pseudopodia that lacked nuclei (Fig. 1B, inset). Staining with ethidium homodimer showed that 17% ±14% of cells at aggregate tips were non-viable, while few dead cells were observed near aggregate bases. Since the vast majority of cells are viable under *P_base_*>*P_tip_*, and since reverting to a control pressure profile allowed multicellular invasions to emerge (*p* = 0.0003; Fig. 2E), it is unlikely that the pressure-induced switch in invasive phenotype resulted from cell death. Likewise, switching an invasive aggregate from a control to elevated *P_base_* profile blocked the elongation of existing invasion(s) and the development of new ones (*p* = 0.002; Fig. 2E). Thus, the invasive phenotype at the aggregate tip responded dynamically to changes in pressure at the aggregate base.

### Directionality of the Pressure Difference Controls the Invasive Response

To determine whether the invasive switch responded purely to a pressure difference or whether the direction of the difference was also important, we subjected aggregates to elevated pressure at the other end of the collagen gel, so that *P_base_*<*P_tip_* and with |*P_base_* − *P_tip_*| ranging from 0.4 to 1.6 cm H_2_O. Compared to the control pressure profile, here the frequency of invasion was greater (*p* = 0.01; Fig. 2A), the invasions contained more cells (*p* = 0.0002; Fig. 2B), and the invasions extended further into the gel (*p*<0.0001; Fig. 2C). Again, the invasions were non-uniformly distributed, with enrichment near the aggregate tip (Fig. 2D).

We did not observe any consistent correlation between the magnitude of the pressure difference |*P_base_* − *P_tip_*| (up to 1.6 cm H_2_O) and invasion frequency (Fig. 2F). In fact, segregation of the *P_base_* ≈ *P_tip_* samples into those for which *P_base_* ♦ *P_tip_* and those for which *P_base_* 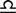 *P_tip_* revealed markedly different behaviors between the two pressure conditions (*p*<0.0001; Fig. 2A, inset). Suppression of invasion required pressure profiles that resulted in a negative base-to-tip pressure gradient, whether transient (as in the case of *P_base_* ♦ *P_tip_*) or sustained (as in *P_base_*>*P_tip_*). The direction, rather than the magnitude, of the pressure difference was the important parameter; even very small changes in pressure were sufficient to affect invasion, as long as the direction of the gradient changed. Altogether, these data suggested that changes in the pressure profile did not suppress invasive phenotype via a direct effect of pressure changes per se, but instead acted via directional changes (e.g., in interstitial flow).

### Pressure-induced Changes in Invasive Phenotype Result from Convective Signals

In theory, the changes in invasive protrusions that are induced by pressure can be mediated through changes in the local chemical and/or mechanical environment at the aggregate tip. For instance, a switch in the direction of the pressure difference could alter the convective transport of cell-derived factors. Alternatively, the pressure profile could control the polarization of the mechanical stresses experienced by cells at the tip. To distinguish between these and other possibilities, we formed aggregates in different configurations that enabled independent variation of the chemical and mechanical changes that were induced by pressure.

**Figure 3:**
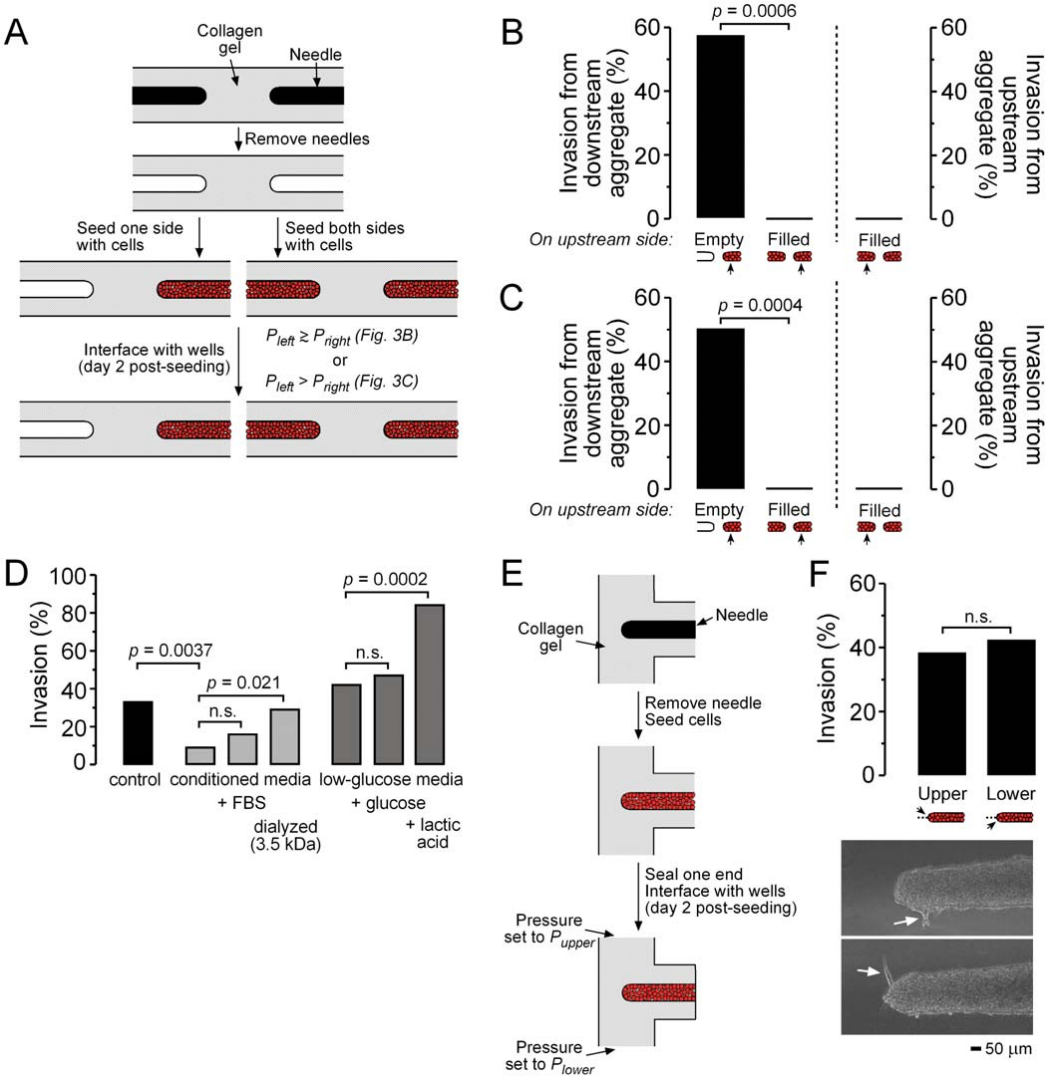
Modulation of invasion via pressure-induced changes in the local chemical microenvironment. (A) Schematic diagram of the formation of opposing aggregates. (B) Invasion frequencies in the presence of an opposing aggregate (*n* = 14) or cavity (*n* = 21) with *P_left_* ♦ *P_right_*. (C) Invasion frequencies in the presence of an opposing aggregate (*n* = 19) or cavity (*n* = 18) with *P_left_*>*P_right_*. (D) Invasion frequencies in single aggregates that were fed with conditioned media and its variants and with *P_base_* 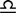 *P_tip_* (*n* = 51, 53, 43, 34, 48, 47, and 32 for control media, conditioned media, conditioned media with 10% FBS, dialyzed conditioned media, low-glucose media, low-glucose media that was supplemented with glucose, and low-glucose media that was supplemented with lactic acid, respectively). (E) Formation of aggregates in a T-shaped gel. (F) *Top*, invasion frequencies at the upstream and downstream halves at the aggregates (*n* = 45). *n.s.*, not significant. *Bottom*, representative images of invasions (arrows). Flow is from top to bottom, perpendicular to the aggregate axes.

By using gels that contained two blind-ended cavities, we formed paired aggregates of cells that had opposing tips (Fig. 3A). This configuration allowed the tips to be subjected to cell-conditioned media, even under control pressure profiles. We found that the presence of an adjacent aggregate mimicked the effect of elevated *P_base_* by eliminating invasion [*p* = 0.0006 for *P_left_* ♦ *P_right_* (Fig. 3B); *p* = 0.0004 for *P_left_*>*P_right_* (Fig. 3C)]. These results suggested that conditioning of interstitial fluid by the aggregate, and convective transport of this fluid to the tip of the aggregate, could be responsible for pressure-induced changes in invasive phenotype.

We tested this possibility directly by collecting cell-conditioned media from the tips of aggregates under *P_base_*>*P_tip_*, and by applying this media to a separate set of samples under *P_base_* 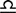 *P_tip_*. Exposure of an aggregate tip to conditioned media reduced invasion (*p* = 0.0037; Fig. 3D). The composition of cell-conditioned media differed appreciably from that of fresh media: the conditioned media had lower levels of glucose (2.3–2.4 mg/mL vs. 3.0 mg/mL) and higher levels of lactic acid (∼0.7 mg/mL (assuming near-quantitative glycolytic efficiency [15]) vs. ∼0 mg/mL). To determine which soluble factor(s) were responsible for the effect of invasion, we also treated aggregates under *P_base_* 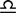 *P_tip_* with fresh media that had the same glucose and/or lactate content as conditioned media. Neither low glucose nor high lactate reproduced the effect of conditioned media; in fact, high lactate led to increased invasion (*p* = 0.0002; Fig. 3D). Surprisingly, dialysis of conditioned media (3.5 kDa cutoff) restored the invasive phenotype (*p* = 0.021; Fig. 3D), whereas supplementation with fetal bovine serum resulted in partial restoration of phenotype that did not reach statistical significance. Thus, a low molecular weight solute that is consumed or produced within the aggregate is likely to be responsible for convective alterations in invasive phenotype.

To determine whether pressure-induced mechanical stresses had any direct effect on invasive phenotype aside from that due to induced convective transport of conditioned media, we also examined the behavior in the upstream aggregate in paired samples. As observed in the downstream aggregate, the upstream one did not invade (Fig. 3B, 3C). Moreover, we formed aggregates in T-shaped collagen gels so that transverse flow could be applied to the aggregate tip (Fig. 3E). Under these conditions, we expect that the tip would be exposed to a constant stream of unconditioned media, and its upstream and downstream halves would be subjected to tensile and compressive flow-induced stresses along the cell-gel interface, respectively. We found that invasion occurred robustly along both sides at the tip (Fig. 3F). Taken together, these data suggest that changes in pressure alter invasive phenotype via changes in convective transport, rather than via a direct mechanical effect.

### Local Solute Concentrations, Rather than Solute Gradients, Mediate Invasive Changes

**Figure 4:**
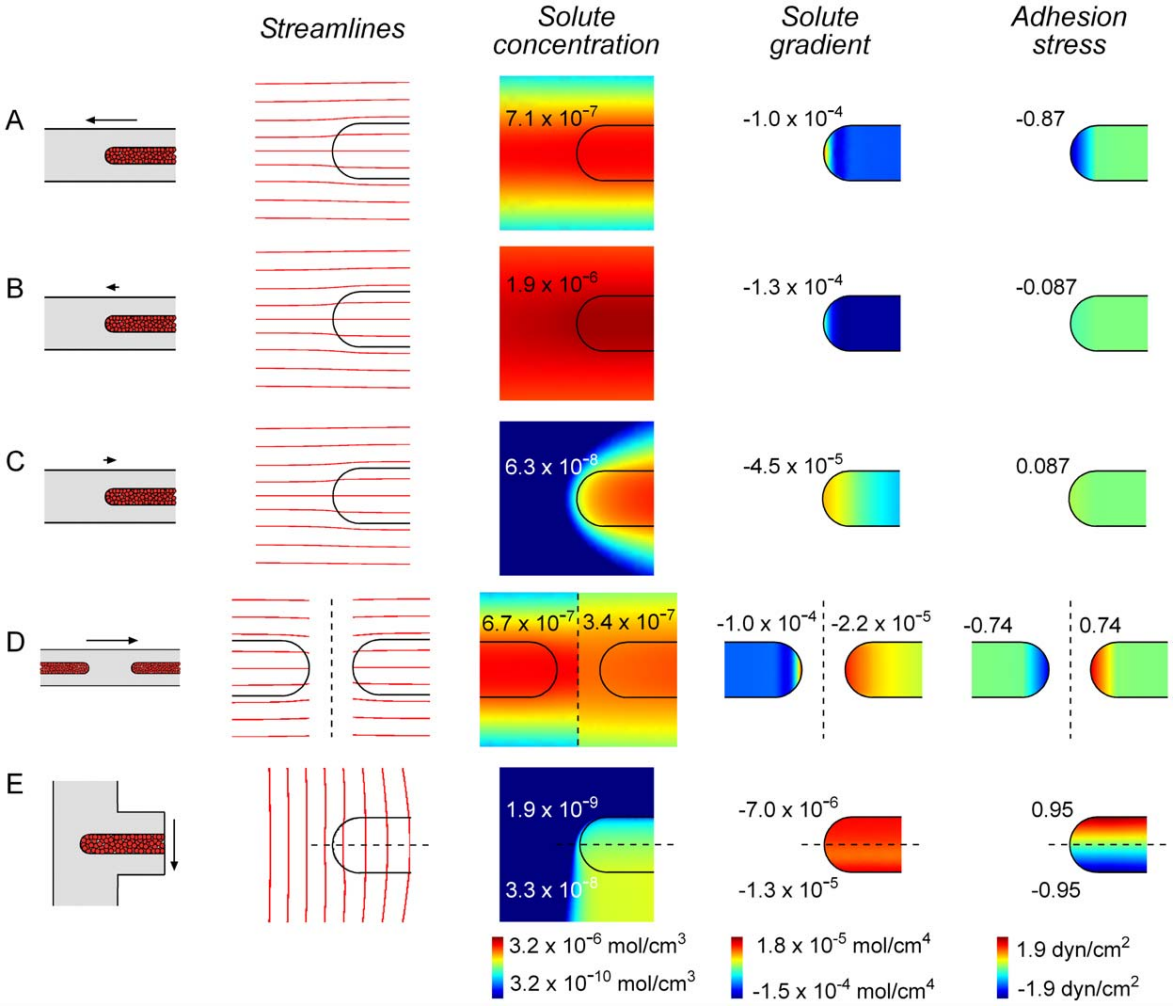
Computational modeling of aggregates under different pressure conditions for a secreted invasion inhibitor. (A–E) Computed concentration, concentration gradient, and flow and stress profiles in single aggregates (A–C), opposing aggregates (D), and aggregates in a T-shaped gel (E). Arrows indicate average interstitial flow velocity. Maps of concentrations use a logarithmic scale; maps of concentration gradients and adhesion stresses use a linear scale. Numbers indicate the average values within 90 µm of the tips of the aggregates (A–D) and at the upstream and downstream halves of the tips (E). In all models, the solute diffusion coefficient was 1 µm^2^/s, and the solute production rate was 3×10^−10^ mol/cm^3^/s.

To determine whether it is the absolute value or gradient of solute concentration that is responsible for pressure-induced changes in invasive phenotype, we solved computational models of fluid and solute transport in the aggregates under the various experimental configurations (Fig. 4, S1). These models yielded the concentration profile of a hypothetical secreted invasion inhibitor (Fig. 4) or consumed invasion promoter (Fig. S1), and the interstitial flow profile in the aggregate and surrounding gel. From the flow at the surface of the aggregate, we also calculated the flow-induced stress exerted at the cell-gel interface [12]. These models required measurement of the hydraulic permeabilities of the collagen gel and cell aggregates, which we found to be (1.1±0.1)×10^−7^ cm^4^/dyn·s and (6.6±3.6)×10^−8^ cm^4^/dyn·s, respectively.

Comparison of computational and experimental results at the aggregate tips showed that pressure-induced changes in absolute solute concentration, rather than in concentration gradient or in cell-gel adhesion stress, were consistent with observed changes in invasion. For secretion of an invasion inhibitor, configurations that inhibited invasion (Fig. 4A, 4B, 4D) resulted in at least a five-fold greater inhibitor concentration at the aggregate tips than in configurations that allowed invasion (Fig. 4C, 4E). Concentration gradients and adhesion stresses, however, did not correlate well with invasive phenotype. For instance, the computed gradient at the downstream side of paired aggregates, a non-invasive site, was similar to those in cell configurations that led to invasion (Fig. 4D). Moreover, aggregates in T-shaped gels experienced positive and negative flow-induced adhesion stresses (Fig. 4E), but were uniformly invasive (Fig. 3F). Models of a secreted invasion promoter yielded similar conclusions (Fig. S1).

## Discussion

Our results show that the invasive phenotype of a model tumor cell aggregate depends on the pressures imposed across the extent of the aggregate. Elevated pressure at the base of an aggregate resulted in convective flow of solutes; when coupled with cell metabolism (either consumption or production of soluble factors in the interstitial fluid), this flow induced a switch in the local chemical microenvironment near the aggregate tip, which led to inhibition of invasion.

### Mediators of Pressure-induced Changes in Invasion

#### Soluble factors

Tumor interstitial fluid varies appreciably from its normal counterpart in chemical composition [16]. Of the major changes, hypoxia [17], [18], acidosis [19], production of lactate [20], and depletion of glucose [17] have all been claimed to result in enhanced invasiveness. In our model, lactic acid robustly increased invasion (Fig. 3D), whereas depletion of glucose did not. Despite these effects of individual solutes, cell-conditioned media as a whole inhibited invasion. Since the invasive phenotype is restored by dialyzing the conditioned media (which should also restore dissolved oxygen content to ambient levels), but not by supplementing with serum, the relevant solute most likely is already present in the DMEM/F12 base media or is secreted by MDA-MB-231 cells as a low molecular weight factor.

These results imply that the degree to which changes in pressure alter invasion depends on two dimensionless parameters, the Péclet number *Pe* (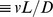) and the Thiele modulus Φ (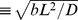). Here, *v* is the interstitial fluid velocity, *L* is the length of the aggregate, *D* is the diffusion coefficient of the relevant solute, and *b* is the rate at which the solute is consumed or produced. The higher the Péclet number and Thiele modulus, the stronger the effect of convection and solute consumption or production, and the larger the switch induced by pressure. Rough estimates for *Pe* and Φ suggest that both quantities are large enough to be consistent with the proposed mechanism of convection and reaction: Under elevated *P_base_* conditions, *v* is on the order of 1 µm/s, *Pe* ≈ 4 for a freely diffusing solute the size of glucose, and Φ ≈ 2.5 for a solute consumed at the same rate as glucose (*b* ≈ 0.0004/s). The effective diffusion coefficient can be much lower if the solute is larger than glucose or if it binds to a carrier protein or cell-surface receptor, and *Pe* and Φ will be correspondingly larger. The identity of this solute remains to be determined.

#### Interstitial flow and mechanical stress

Recent studies have shown that interstitial flow is an important modulator of cell invasion [7], [21]. In low-density cultures of human breast cancer cells, interstitial flow induces downstream migration via “autologous chemotaxis”, in which flow polarizes the local concentration of an autocrine chemokine [5]. In high-density cultures, migration may occur upstream, possibly via polarization of integrin-mediated mechanotransduction [7]. In our model system, convection-induced changes in chemical composition alone appeared to be sufficient to account for the observed effects of elevated pressure. Using opposing or T-shaped aggregates, we found that the chemical microenvironment dominated any purely mechanical effects (Fig. 3B, 3C, 3F). We note that it may be possible to alter the relative contributions of chemical and mechanical signals to invasion by modifying the sizes of the aggregates. Autologous chemotaxis is unlikely to explain our findings, since the migration in aggregates was upstream, not downstream, and since computational models showed that invasion correlated better with solute concentrations than with concentration gradients (Fig. 4, S1).

We observed that aggregates under invasive pressure profiles distended the surrounding gel. The average diameters near the tips were 134.9±14.2 µm and 105.6±9.9 µm for single aggregates under *P_base_* 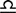 *P_tip_* and *P_base_*>*P_tip_*, respectively. Thus, the tips should experience a compressive stress under invasive pressure conditions. This finding is consistent with a recent study of stress-induced invasion in two-dimensional cultures of 67NR mammary epithelial cells [6]. Thus, one possible mechanism by which elevated pressure inhibits invasion may be a reduction of aggregate diameter (via chronic exposure to cell-conditioned media) and a resulting decrease in external compression.

#### Matrix organization

Several studies have shown that the microscale organization of the tumor matrix can control the extent of invasion [4], [22], [23]. Increases in collagen density can promote invasion, in part via integrin signaling [4], [23]. The local orientation of collagen fibers at the interface between a tumor and the surrounding tissue can also modulate the invasive phenotype; the presence of circumferentially- and radially-oriented fibers correlate with weak and strong invasion, respectively [22].

We have shown previously that molding collagen, by itself, does not lead to visible heterogeneities by confocal reflectance microscopy [24]. In our aggregates, one might expect changes in local organization of the surrounding collagen gel as the population of cells grows to partially account for changes in invasion. For instance, having *P_base_*>*P_tip_* could lead to compaction of collagen at the surface of the aggregate as the cells are “pushed” into the gel and thus to inhibition of invasion. The smaller diameter of the aggregates under this pressure condition (see above), however, suggests it is unlikely that collagen compaction is responsible for inhibition of invasion in this system. We cannot rule out the possibility that other microscale changes in collagen organization play a role in pressure-induced changes in invasion.

### Potential Implications

By in vivo standards, the pressures used in this study are low (on the order of 1 cm H_2_O versus 10 cm H_2_O in tumors [25]). The resulting pressure gradients are also low (on the order of 1 cm H_2_O/cm versus 100 cm H_2_O/cm in vivo [26]). The hydraulic permeabilities of the collagen gel and cell aggregate, however, are orders-of-magnitude larger than those of normal and tumor tissues in vivo [27], so the resulting interstitial flow velocities of ∼1 µm/s here are similar to those observed in vivo [28]. Since our proposed mechanism for pressure-induced suppression of invasion depends on interstitial convection, it is conceivable that similar phenomena may be present in vivo. Indeed, the centrifugal flows present in tumors in vivo are sufficiently large to export therapeutic agents from the tumor core to the rim and thereby limit the residence times of these agents [29].

One must keep in mind that the aggregates we studied here are only model systems, as they lack the heterogeneous epithelial and stromal cell populations that comprise actual tumors; the addition of tumor stromal cells may enhance [30] or retard [31] invasion. It will be interesting to see if interstitial fluid from clinical and experimental tumors alter tumor cell invasion and, if so, to determine the cell type(s) responsible for this activity. More broadly, our work suggests that changes in pressure can induce non-local changes in invasive phenotype in a tumor cell aggregate, and adds to the concept that physical signals can affect the behavior of tumor cells.

## Supporting Information

Figure S1 **Computational modeling of aggregates under different pressure conditions for a consumed invasion promoter.** (A–E) Computed concentration and concentration gradient profiles in single aggregates (A, B, C), opposing aggregates (D), and aggregates in a T-shaped gel (E). Arrows indicate average interstitial flow velocity. Maps of concentrations and concentration gradients use a logarithmic and linear scale, respectively. Numbers indicate the average values within 90 µm of the tips of the aggregates (A–D) and at the upstream and downstream halves of the tips (E). In all models, the solute diffusion coefficient was 1 µm^2^/s, and the solute consumption rate constant was 1/300 s^−1^.
(TIF)
