# Supplementary figures and images for: Modulation of Invasive Phenotype by Interstitial Pressure-Driven Convection in Aggregates of Human Breast Cancer Cells
Source: PLoS One. 2012 Sep 18;7(9):e45191. doi: 10.1371/journal.pone.0045191 (PMC3445465; doi:10.1371/journal.pone.0045191)

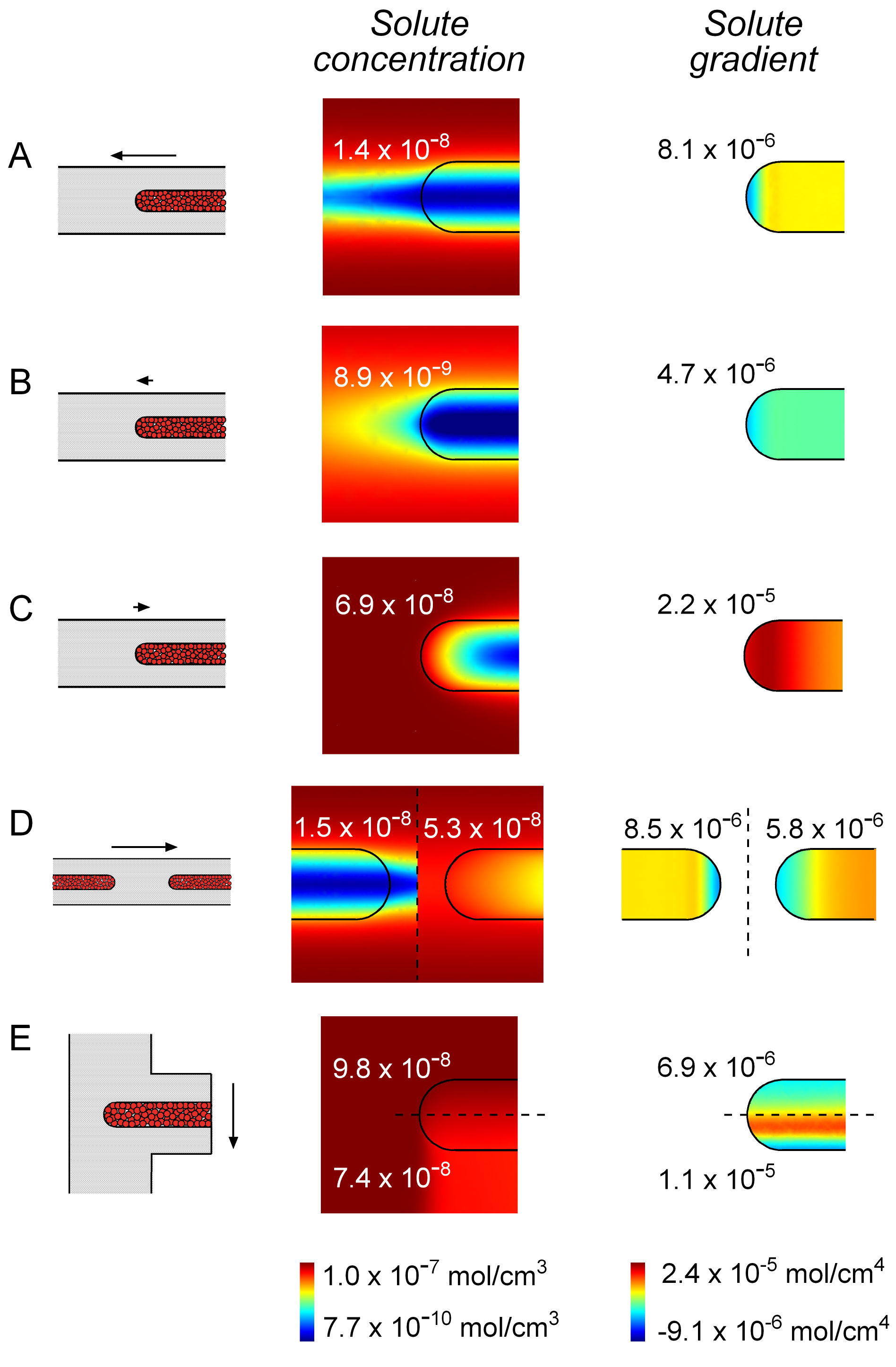

Supplement: Figure S1 — Computational modeling of aggregates under different pressure conditions for a consumed invasion promoter. (A–E) Computed concentration and concentration gradient profiles in single aggregates (A, B, C), opposing aggregates (D), and aggregates in a T-shaped gel (E). Arrows indicate average interstitial flow velocity. Maps of concentrations and concentration gradients use a logarithmic and linear scale, respectively. Numbers indicate the average values within 90 µm of the tips of the aggregates (A–D) and at the upstream and downstream halves of the tips (E). In all models, the solute diffusion coefficient was 1 µm2/s, and the solute consumption rate constant was 1/300 s−1. (TIF) [file pone.0045191.s001.tif]
